# Characterization of *Fusarium verticillioides* Med1 LxxLL Motif Involved in Fumonisin Biosynthesis

**DOI:** 10.3390/toxins15110652

**Published:** 2023-11-13

**Authors:** Zehua Zhou, Jie Liu, Jie Zhang, Huijuan Yan, Tuyong Yi, Won Bo Shim

**Affiliations:** 1 Hunan Provincial Key Laboratory for Biology and Control of Plant Pests, Hunan Agricultural University, Changsha 410128, China; m15895982813@163.com (Z.Z.); 13618426557@stu.hunau.edu.cn (J.L.); 2 College of Plant Protection, Nanjing Agricultural University, Nanjing 210095, China; 2018202061@njau.edu.cn; 3 Department of Microbiology and Immunology, University of California San Francisco, San Francisco, CA 94143, USA; huijuan.yan@ucsf.edu; 4 Department of Plant Pathology and Microbiology, Texas A&M University, College Station, TX 77843, USA

**Keywords:** *Fusarium verticillioides*, fumonisin B_1_, Med1 LxxLL motif, starch metabolism, carotenoids biosynthesis

## Abstract

The Med1 transcriptional coactivator is a crucial component of the Mediator middle complex, which regulates the expression of specific genes involved in cell development, differentiation, reproduction, and homeostasis. The Med1 LxxLL motif, a five-amino-acid peptide sequence, is essential for Med1-mediated gene expression. Our previous study revealed that the disruption of the Med1 subunit leads to a significant increase in fumonisin B_1_ (FB_1_) production in the maize pathogen *Fusarium verticillioides*. However, our understanding of how Med1 regulates FB_1_ biosynthesis in *F. verticillioides*, particularly through the Med1 LxxLL motifs, remains limited. To characterize the role of LxxLL motifs, we generated a series of Med1 LxxLL deletion and amino acid substitution mutants. These mutants exhibited impaired mycelial growth and conidia germination while demonstrating enhanced conidia production and virulence. Similar to the Med1 deletion mutant, Med1 LxxLL motif mutants also exhibited increased FB_1_ biosynthesis in *F. verticillioides*. Proteomic profiling revealed that the Med1 LxxLL motif regulated the biosynthesis of several key substances that affected FB_1_ production, including starch and carotenoid. Subsequent studies demonstrated that the production of amylopectin, which is strongly linked to FB_1_ biosynthesis, was significantly increased in Med1 LxxLL motif mutants. In addition, the disruption of carotenoid metabolic genes decreased carotenoid content, thus stimulating FB_1_ biosynthesis in *F. verticillioides*. Taken together, our results provide valuable insights into how the Med1 LxxLL motif regulates FB_1_ biosynthesis in the mycotoxigenic fungus *F. verticillioides*.

## 1. Introduction

The Mediator complex, also known as Mediator, is a large multi-subunit protein complex that is indispensable for the regulation of RNA polymerase II (Pol II) activity [1,2]. Mediator was first purified from *Saccharomyces cerevisiae* and was proven critical for transcriptional regulation [3,4]. The organization and structure of the Mediator are conserved within eukaryotes, with four functional modules: Head, Middle, Tail, and Kinase [5,6,7]. Significantly, the Med14 subunit acts as a scaffold to hold together the “core Mediator”, which includes the Head, Middle, and Tail modules [8,9,10,11]. The Kinase module binds reversibly with the “core Mediator”, thereby regulating transcription [6,12,13,14].

The Med1 subunit is considered a key transcription coactivator in the Mediator complex. A previous study showed that the Med1 null mutation was embryonically lethal at midgestation in mice, indicating that Med1 was critical for embryonic development [15]. Jia and colleagues reported that Med1 deficiency led to the disturbance of peroxisome proliferator-activated receptor α (PPARα) target gene transcription and the abrogation of peroxisome proliferation in liver parenchymal cells [16]. In addition, Med1 was indispensable for the expression of genes regulated by the constitutive androstane receptor (CAR) in mouse livers [16]. Furthermore, Med1 was identified as an integrator of multiple transcriptional pathways, including those related to lipid metabolism and hepatic autophagy in HepG2 cells [17].

Protein-protein interactions involved in the regulation of transcription, translation, and cell signaling pathways are dependent on short peptide-recognition motifs, which include the polyproline α-helix and the array of peptide sequences [18,19]. The Leu-Xaa-Xaa-Leu-Leu (LxxLL) motif, one of the important peptide sequences, was first discovered in cofactor proteins that bind with hormone-activated nuclear receptors (NRs) [20]. In addition, Med1 LxxLL motifs have also been observed in non-nuclear receptors, including calcium response element-binding (CREB) protein-binding protein, p300, transcription factors, and Mediator subunits [21,22,23]. Human Med1 contains two LxxLL motifs, which are required for the interaction between Med1 and various NRs. These receptors are involved in the regulation of cellular proliferation, differentiation, and metabolism (energy, lipid, etc.) [16,17,24,25,26]. Jiang and colleagues showed that Med1 LxxLL motifs were indispensable for pubertal mammary gland development and luminal cell differentiation in mice [27]. In addition, Med1 LxxLL motifs contributed to the interaction between Med1 and the glucocorticoid receptor (GR), thereby regulating GR-mediated transcription [25]. Although the Med1 subunit and its LxxLL motif have been well studied in humans and mammals, our understanding of their biological functions in phytopathogenic fungi is quite limited.

Maize (*Zea mays*) is one of the most important cereal crops worldwide. It is a commodity that yields a wide range of human foods, animal feeds, and industrial products. In China, corn production was ranked first among all cereal crops, with 43.07 million hectares planted and 277 million tons harvested in 2022, followed by rice (*Oryza sativa*) and wheat (*Triticum aestivum*) (China-NBS, 2023). *Fusarium verticillioides* is a major fungal pathogen of maize that causes diseases such as maize ear rot and stalk rot, leading to yield losses. Significantly, *F. verticillioides* produces mycotoxins on infested corn and renders it unacceptable for human food products and animal feedstuffs, depending on the contamination level. Among various mycotoxins produced by *F. verticillioides*, fumonisin B_1_ (FB_1_) is the most frequently detected form and poses a particular concern, with links to liver damage, embryo development defects, and esophageal cancer in humans and other diseases in livestock [28,29,30,31,32]. While the fumonisin biosynthesis gene cluster has been identified in the *F. verticillioides* genome [33], the mechanism of how the fungus regulates fumonisin biosynthesis when infesting maize remains elusive. Therefore, gaining a deeper understanding of the regulatory network in fumonisin biosynthesis will assist in developing effective management strategies to reduce mycotoxin contamination in food and feedstuffs.

Our previous study learned that the Med1 deletion mutation significantly increased FB_1_ biosynthesis in *F. verticillioides* [34]. In this study, we identified one LxxLL motif in the *F. verticillioides* Med1 protein and showed that mutations in the Med1 LxxLL motif upregulate the expression of Fum8, thereby leading to increased FB_1_ production. We implemented proteomic analysis and gene knockout experiments to determine how the Med1 LxxLL motif regulates FB_1_ biosynthesis in *F. verticillioides*. Our results showed that the Med1 LxxLL motif participates in the regulation of mitochondrial function and cellular metabolism. Significantly, starch metabolism and carotenoid biosynthesis were affected by Med1 LxxLL motif mutations, which negatively impact FB_1_ biosynthesis. This study provided a deeper molecular characterization of how the Med1 LxxLL motif plays a critical regulatory role in fumonisin biosynthesis in the mycotoxigenic fungus *F. verticillioides*.

## 2. Results

### 2.1. Identification of Med1 LxxLL Motif in F. verticillioides

**Figure 1:**
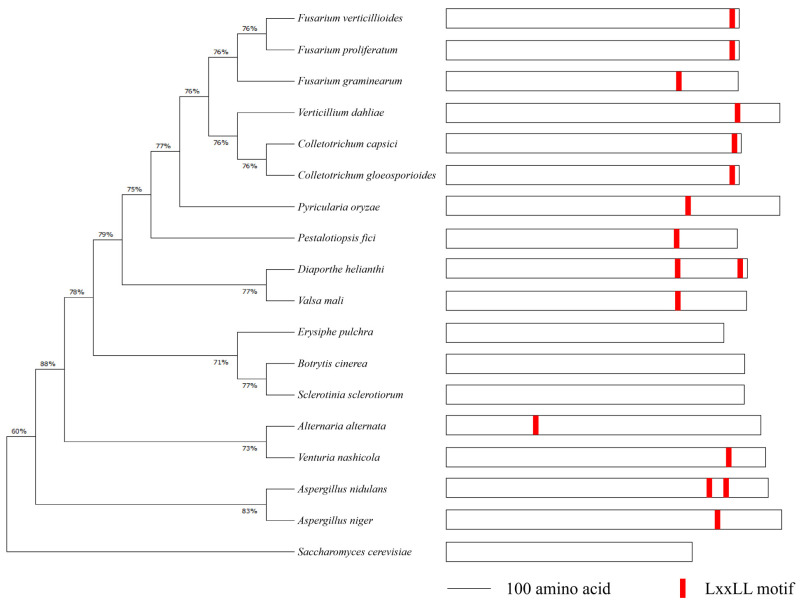
Phylogenetics of Med1 homologs and LxxLL motif analysis in *F. verticillioides* (FVEG_02872) and other 18 representative fungal species. The alignment was performed with the MEGA program, version 4.0. The tree was produced via the maximum-likelihood method. The LxxLL motifs were indicated with a red orthogon.

To identify the Med1 LxxLL motif throughout phytopathogenic fungi, we retrieved the protein sequences of Med1 homologs in 18 representative fungal species, including *F. verticillioides*, *F. graminearum*, *Botrytis cinerea*, *Sclerotinia sclerotiorum*, *Aspergillus nidulans*, *Colletotrichum capsici*, and *S. cerevisiae*, from the NCBI database for sequence analysis. The phylogenetic tree showed that Fusarium species shared a high Med1 subunit homology (Figure 1, left panel). We found that *F. verticillioides* and other 13 fungal species contained at least one LxxLL motif in the Med1 protein. Among the 14 fungal species, *A. nidulans* and *Diaporthe helianthi* contained two Med1 LxxLL motifs (Figure 1, right panel). However, no Med1 LxxLL motif was found in *S. cerevisiae*, *B. cinerea*, *S. sclerotiorum*, or *Erysiphe pulchra* (Figure 1). These results suggested that FvMed1 and its LxxLL motif hold distinct characteristics from Med1 homologs in other phytopathogenic fungi.

### 2.2. Med1 LxxLL Motif Plays a Role in F. verticillioides Development and Virulence

**Figure 2:**
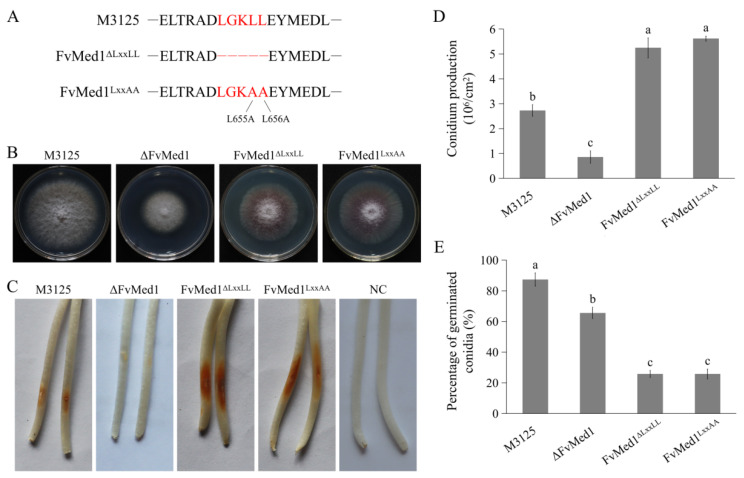
The Med1 LxxLL motif is involved in fungal development processes and virulence in *F. verticillioides*. (**A**) Construction of FvMed1 LxxLL deletion and amino acid substitution mutants. The red letters in up, middle and down panels indicated the amino acid in wild-type M3125, deletion of amino acid “LGKLL” and amino acid substitutions of “LGKAA”, respectively. (**B**) Colony morphology of the wild-type M3125, ΔFvMed1, FvMed1^ΔLxxLL,^ and FvMed1^LxxAA^ mutants on PDA plates at 28 °C for 5 days. (**C**) The impact of FvMed1 LxxLL mutations on virulence. Maize seedlings were grown in the dark for 3 days and then inoculated with the conidia suspension of each mutant. The images were taken after 7 days of inoculation. Sterilized water was used as a negative control. (**D**) Conidial germination of the wild-type M3125 and Med1 LxxLL mutants. The conidia of each strain were cultured on WA plates. After incubation at 28 °C for 7 h, the conidial germination of 200 conidia was examined. (**E**) Conidial production of the wild-type M3125 and Med1 LxxLL mutants. Conidia were collected from the strains cultured on PDA plates at 28 °C for 5 days. Bars denote standard errors from three repeated experiments. The values on the bars followed by the same letter are not significantly different (*p* < 0.05) according to Fisher’s LSD test.

To characterize the functional roles of the Med1 LxxLL motif in *F. verticillioides*, we generated two mutants: FvMed1^ΔLxxLL^, where the motif was deleted, and FvMed1^LxxAA^, where the LxxLL motif was converted to the LxxAA motif through two amino acid substitutions (Figure 2A). The two mutants exhibited a slight growth reduction on potato dextrose agar (PDA) plates after incubation at 28 °C for 5 days when compared to the wild-type strain M3125 (Figure 2B). We found a significant increase in conidia production in FvMed1^ΔLxxLL^ and FvMed1^LxxAA^ when compared to the wild-type M3125 and ΔFvMed1 mutant (Figure 2D). This is important since conidia abundance plays an important role in pathogen transmission and host infection [35,36,37]. However, FvMed1^ΔLxxLL^ and FvMed1^LxxAA^ exhibited a severe defect in conidia germination (Figure 2E). To determine the role of the Med1 LxxLL motif in virulence, conidial suspensions of all mutants were inoculated on maize seedlings as previously described [34,38]. Intriguingly, the mutants FvMed1^ΔLxxLL^ and FvMed1^LxxAA^ exhibited more severe seedling rot symptoms 7 days after inoculation when compared with the M3125 wild-type strain (Figure 2C). These results indicated that the Med1 LxxLL motif is important for fungal development processes and virulence, and we speculated that the increased conidia abundance might overcome the defect of conidia germination in Med1 LxxLL motif mutants during plant infection.

### 2.3. Med1 LxxLL Mutations Significantly Stimulate FB_1_ Biosynthesis and Upregulate Fum8 Gene Expression

**Figure 3:**
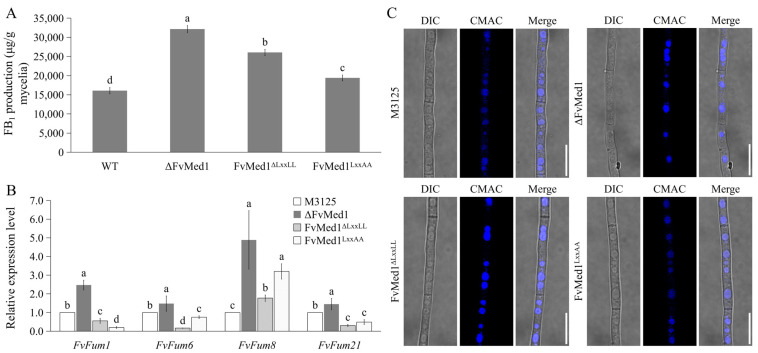
The Med1 LxxLL motif is a suppressor for FB_1_ biosynthesis in *F. verticillioides*. (**A**) The relative expression level of *Fum8* was significantly elevated in Med1 LxxLL mutants when cultured in Myro medium at 28 °C for 2 days. (**B**) FB_1_ production of the wild-type M3125 mutants ΔFvMed1, FvMed1^ΔLxxLL,^ and FvMed1^LxxAA^ in Myro medium at 28 °C for 7 days. (**C**) Vacuole morphology was not affected in Med1 LxxLL mutants. Blue dots in the hyphae represent fungal vacuoles. Bars denote standard errors from three repeated experiments. The values on the bars followed by the same letter are not significantly different (*p* < 0.05) according to Fisher’s LSD test.

Our previous publication showed that deletion of the Med1 subunit significantly increased FB_1_ biosynthesis in *F. verticillioides* [34]. In this study, when cultured in Myro medium for 7 days, FB_1_ production was increased by 62.0% and 20.7% in FvMed1^ΔLxxLL^ and FvMed1^LxxAA^ as compared to M3125, respectively (Figure 3A). In addition, the expression of the Fum8 gene in FvMed1^ΔLxxLL^ and FvMed1^LxxAA^ was increased by 1.8- and 3.2-fold in comparison to M3125, respectively (Figure 3B). One unexpected outcome was that the expression of *Fum1*, *Fum6*, and *Fum21* was repressed in FvMed1^ΔLxxLL^ and FvMed1^LxxAA^, while their expressions were increased in ΔFvMed1 when compared to M3125 (Figure 3B). Our recent study demonstrated the crucial role of vacuoles as an important intracellular organelle in FB_1_ biosynthesis [39]. We hypothesized that deletion of Med1 may alter vacuolar morphology, thereby leading to enhanced FB_1_ biosynthesis. To test this hypothesis, we examined the vacuolar morphology in mutants ΔFvMed1, FvMed1^ΔLxxLL^, and FvMed1^LxxAA^ in the current study. However, all strains exhibited normal round-like vacuoles after being stained with CMAC (Figure 3C), suggesting that the vacuole morphology remained unaffected by the mutations. Taken together, our findings suggested that the regulatory mechanism of the Med1 subunit and its LxxLL motif on FB_1_ biosynthesis was vacuole-independent, which needs further investigation.

### 2.4. Proteomic Profiles of Med1 LxxLL Mutants Grown under Fumonisin-Inducing Conditions

**Figure 4:**
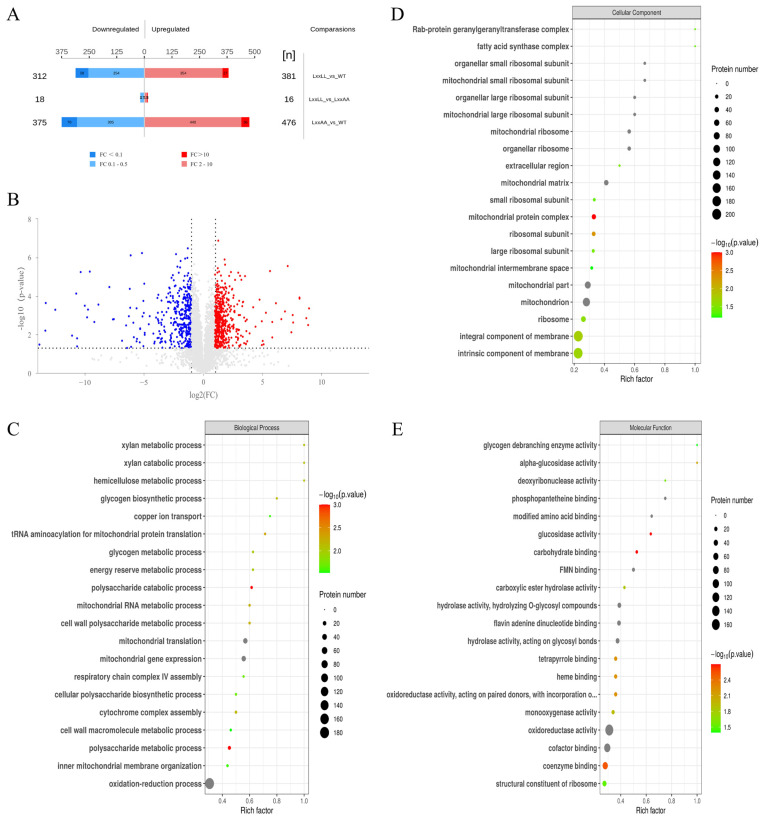
Identification and functional classification of differentially expressed proteins in the wild-type M3125 and Med1 LxxLL motif mutants. (**A**) Numbers of up- and down-regulation proteins. (**B**) Volcano plot showing the differentially expressed proteins between FvMed1ΔLxxLL and wild-type M3125 (upregulation more than two times or downregulation less than 0.5, *p* < 0.05). Red spots represent upregulated proteins, and blue spots represent downregulated proteins. Gray dots represent proteins that showed no significant difference in expression level. Proteins identified from Med1 LxxLL deletion mutant FvMed1ΔLxxLL were classified as “biological process” (**C**), “cellular component” (**D**), and “molecular function” (**E**) according to the GO terms. The *p*-value (*p* < 0.05) was adjusted using the Fisher’s exact test.

To gain further insight into how the Med1 LxxLL motif impacts fumonisin biosynthesis in *F. verticillioides* at the protein level, we performed proteomic analysis with wild-type M3125, FvMed1ΔLxxLL, and FvMed1LxxAA under fumonisin-inducing conditions. In total, we identified 693 and 851 proteins with significantly altered expression (fold change > 2, and *p* < 0.05) in FvMed1^ΔLxxLL^ and FvMed1^LxxAA^, respectively (Figure 4A,B). Noticeably, only 34 different abundant proteins were found between FvMed1^ΔLxxLL^ and FvMed1^LxxAA^, demonstrating the consistency in protein expression under fumonisin-inducing conditions between FvMed1^ΔLxxLL^ and FvMed1^LxxAA^. To characterize the involvement of these differentially expressed proteins (DEPs) in biological functions and processes, GO and KEGG enrichment analyses were conducted. GO enrichment analysis of biological processes (BP) showed that the DEPs were enriched in the oxidation-reduction process, mitochondrial gene expression, and mitochondrial translation (Figure 4C). GO terms enriched in cellular components (CC) and molecular function (MF) found that the DEPs participated in the mitochondrial matrix, mitochondrial ribosome, mitochondrion, oxidoreductase activity, and cofactor binding (Figure 4D,E).

**Figure 5:**
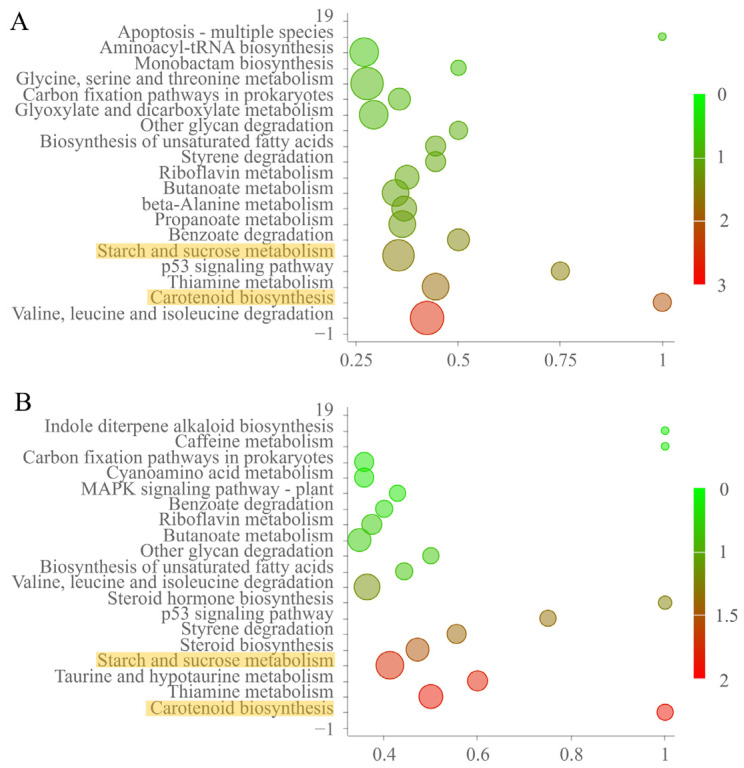
KEGG enrichment analysis of differentially expressed proteins in Med1 LxxLL deletion mutant FvMed1^ΔLxxLL^ (**A**) and Med1 LxxLL amino acid substitution mutant FvMed1^LxxAA^ (**B**) as compared with the wild-type M3125. The abscissa indicates the degree of significant enrichment. The *p*-value (*p* < 0.05) was adjusted using the Fisher’s exact test. The interested pathways are highlighted in yellow.

KEGG enrichment analysis of DEPs revealed that carotenoid biosynthesis, starch and sucrose metabolism, and the thiamine metabolism pathway were significantly enriched in FvMed1^ΔLxxLL^ and FvMed1^LxxAA^ (Figure 5). These results indicated that the Med1 LxxLL motif is involved in the regulation of mitochondrial function and cellular metabolism under fumonisin-inducing conditions in *F. verticillioides*.

### 2.5. Med1 LxxLL Motif Regulates Amylopectin Production in F. verticillioides

**Figure 6:**
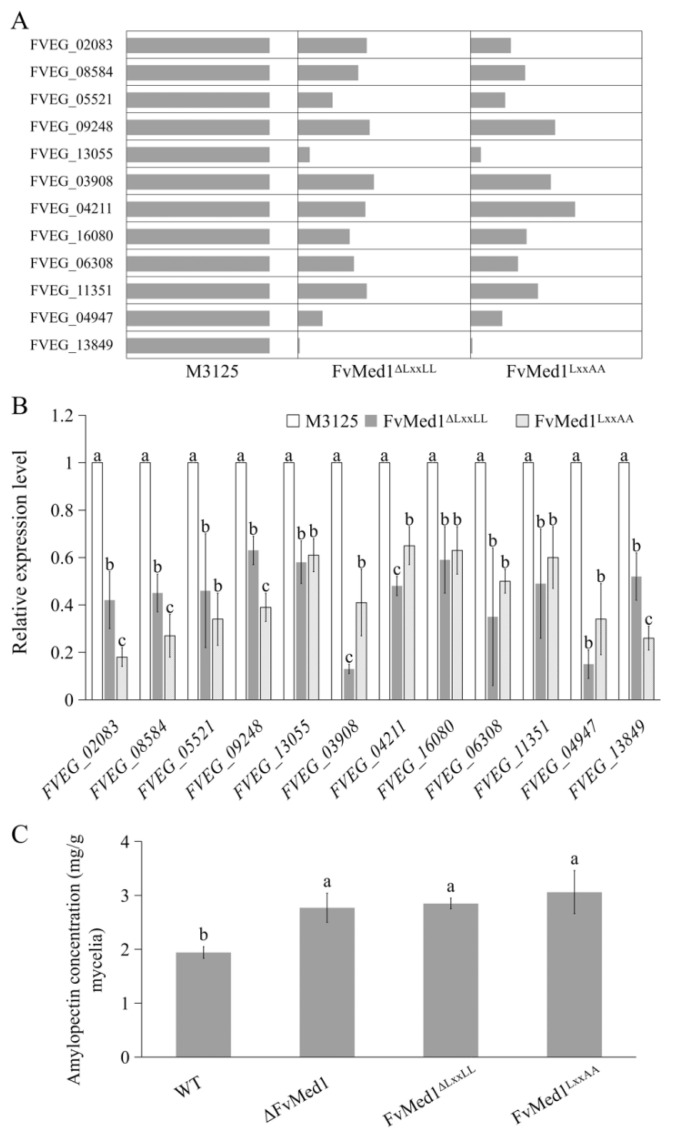
Starch metabolism is regulated by the Med1 LxxLL motif in *F. verticillioides*. Expression levels of key proteins involved in the starch metabolism pathway were significantly decreased in Med1 LxxLL mutants as compared with the wild-type M3125 based on (**A**) proteomic data and (**B**) qRT-PCR assays. (**C**) Mutations in the Med1 LxxLL motif led to the accumulation of amylopectin in *F. verticillioides*. Bars denote standard errors from three repeated experiments. The values on the bars followed by the same letter are not significantly different (*p* < 0.05) according to Fisher’s LSD test.

Previous studies have demonstrated that amylopectin, one of the key polysaccharide components in maize, could increase FB1 accumulation in both maize kernels and liquid medium [40,41]. In the current study, KEGG enrichment analysis showed that the DEPs were enriched in the starch metabolism pathway. Specifically, we observed that 12 proteins were significantly downregulated in both FvMed1^ΔLxxLL^ and FvMed1^LxxAA^ in this pathway (Figure 6A). To further verify the proteomic data, the transcription levels of 12 starch metabolic genes were validated by qRT-PCR assays. We found that all tested genes were significantly suppressed in Med1 LxxLL motif mutants (Figure 6B). Furthermore, we measured amylopectin content in the mycelia of FvMed1^ΔLxxLL^ and FvMed1^LxxAA^ with an Amylopectin Assay Kit (Solarbio, Beijing China). As shown in Figure 6C, amylopectin content in FvMed1^ΔLxxLL^, FvMed1^LxxAA,^ and ΔFvMed1 was significantly higher than that in M3125. These results suggested that the enhanced FB_1_ biosynthesis in Med1 LxxLL motif mutants was associated with starch metabolism and amylopectin accumulation in *F. verticillioides*.

### 2.6. Suppressed Carotenoid Production Contributed to Enhanced FB_1_ Biosynthesis in Med1 LxxLL Mutants

**Figure 7:**
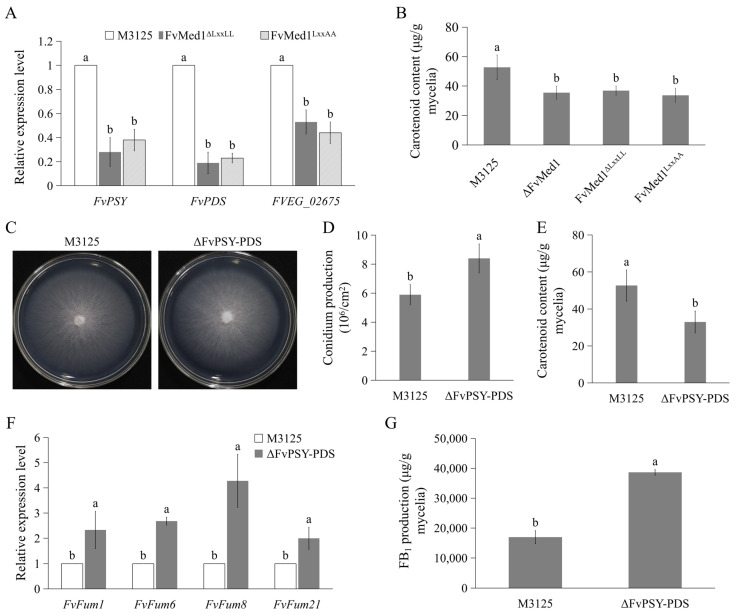
A reduction in carotenoid production caused by Med1 LxxLL mutations accelerates FB_1_ biosynthesis in *F. verticillioides*. (**A**) Expression levels of key genes associated with carotenoid biosynthesis were drastically decreased in Med1 LxxLL mutants compared to the wild-type M3125. (**B**) Mutations in the Med1 LxxLL motif significantly suppressed carotenoid production in *F. verticillioides*. (**C**) Colony morphology of the wild-type M3125 and mutant ΔFvPSY-PDS on PDA plates at 28 °C for 5 days. (**D**) The disruption of carotenoid biosynthesis increased conidial production in *F. verticillioides*. Conidia were collected from the strains cultured on PDA plates at 28 °C for 5 days. (**E**) Carotenoid content in the wild-type M3125 and the mutant ΔFvPSY-PDS. (**F**) The relative expression level of fumonisin biosynthetic genes was significantly elevated in mutant ΔFvPSY-PDS when cultured in Myro medium at 28 °C for 2 days. (**G**) The mutant ΔFvPSY-PDS exhibited increased FB_1_ production as compared with the wild-type M3125 in Myro medium. After growth in Myro for 7 days, all strains were determined for FB_1_ production. Bars denote standard errors from three repeated experiments. The values on the bars followed by the same letter are not significantly different (*p* < 0.05) according to Fisher’s LSD test.

When analyzing proteomic profiles, we also found that mutations in Med1 LxxLL resulted in significant enrichment proteins associated with carotenoids metabolism (Figure 5). The expression of *FvPSY*, *FvPDS*, and *FVEG_02675* was confirmed by qRT-PCR assays. The results showed that the expression of these genes was significantly decreased in Med1 LxxLL mutants (Figure 7A). Furthermore, we assessed the content of carotenoids in the mycelia of both M3125 and Med1 LxxLL mutants. In line with proteomic results, the disruption of the Med1 or Med1 LxxLL motif significantly decreased carotenoid production in *F. verticillioides* (Figure 7B). To characterize the roles of carotenoids in FB1 production, we generated a mutant strain, ΔFvPSY-PDS, by deleting the coding regions of FvPSY and FvPDS in *F. verticillioides*. The mutant exhibited no obvious defects in vegetative growth on the PDA plate but a significant increase in conidiation compared to M3125 (Figure 7C,D). As expected, the production of carotenoids was significantly inhibited in ΔFvPSY-PDS when cultured in Myro medium for 2 days (Figure 7E). Moreover, the expression level of fumonisin biosynthetic genes, including *Fum1*, *Fum6*, *Fum8*, and *Fum21*, was significantly enhanced (Figure 7F). In addition, FB1 production was increased by 2.3-fold in ΔFvPSY-PDS when compared to M3125 (Figure 7G). Taken together, these findings suggest that the reduced production of carotenoids in Med1 LxxLL mutants contributes to enhanced FB_1_ biosynthesis.

## 3. Discussion

The Mediator complex is a conserved multi-subunit complex that plays important roles in the regulation of RNA polymerase II activity [1,2]. The protein complex is implicated in almost all transcriptional activities across eukaryotes [1,42,43]. In addition, the Mediator contributes to considerable physiological processes, and its dysregulation leads to a variety of disorders, including cell viability defects, embryonic lethality, and multiple cancers [1,6,44]. The Mediator is composed of at least 25–30 subunits and divided into four modules based on biochemical, genetic, and structural evidence [6,7,34]. It has been demonstrated that different Mediator subunits are involved in regulating distinct subsets of genes. In yeast, the Med17 and Med2 subunits were required for transcription of almost all protein-coding genes [12,45]. In the human pathogenic fungus *Candida albicans*, Ssn3 and Ssn8 were determined to be indispensable for the regulation of glucose metabolism and biofilm formation [46]. In addition, the Med31 subunit was found to interact with SCARECROW protein, a key regulator of plant stem cell asymmetrical division, thereby orchestrating radial patterning of roots in *Arabidopsis thaliana* [47].

The Med1 subunit, a key transcriptional coactivator in the Mediator middle module, is critical for mediating the interactions between Mediator and NRs [48]. In human cells, Med1 is required for glucocorticoid receptor-regulated gene transcription [25]. In addition, the Med1 subunit was indispensable for optimal constitutive androstane receptor-mediated gene expression in mice [16]. While the biological functions of Med1 in humans and mammals are well studied, the understanding of Med1 in phytopathogenic fungi is limited. Our recent publication showed that Med1 participates in fungal development processes and plant infection. Interestingly, while most of the Mediator subunit deletion mutants exhibited defects in fumonisin biosynthesis, we found significantly increased accumulation in the Med1 deletion mutant [34]. This finding suggested a unique role of Med1 in FB_1_ biosynthesis compared to other subunits. However, the underlying regulatory mechanism of Med1 in FB_1_ biosynthesis is not clearly understood.

Med1 LxxLL motifs play a crucial role in facilitating interactions between NRs and Med1 [24,49]. When using reconstituted Mediator containing Med1 LxxLL motif mutations, studies demonstrated that these motifs are important for the interactions of NRs with Mediator, thus optimizing NR-mediated transcriptions [27,50,51]. Jiang and colleagues reported that Med1 LxxLL motifs are indispensable for mammary epithelial cell differentiation in mice [27]. In addition, it was found that the two Med1 LxxLL motifs contribute equally to the optimal interactions between Med1 and GR [25]. Unfortunately, the understanding of how Med1 functions in plant pathogens is far from clear. In this study, two forms of Med1 LxxLL motif mutants (FvMed1^ΔLxxLL^ and FvMed1^LxxAA^) were constructed. We determined that the Med1 LxxLL motif is involved in fungal development processes and virulence. In addition, the mutations of the Med1 LxxLL motif significantly increased FB_1_ production in *F. verticillioides*. We also found that the expression of *Fum1*, *Fum6*, *Fum8*, and *Fum21* was upregulated in the Med1 deletion mutant. Interestingly, the expression level of *Fum8* was elevated in Med1 LxxLL motif mutants, while the transcription of *Fum1*, *Fum6*, and *Fum21* was downregulated. Published studies showed that Mediator could be recruited to NR target genes through physical interactions with DNA-binding transcription factors that act synergistically with NRs on specific genes [52,53,54] and that the LxxLL motif is vital for the interactions of Med1 and various proteins [27,48,51]. We propose that such interactions may exist in FvMed1 and FvFum8, and that the presence of the LxxLL motif represses the activity of FvFum8 in *F. verticillioides*. Our data raise questions regarding why the Med1 LxxLL motif mutations significantly increase the expression of Fum8, but not the other key fumonisin biosynthetic genes in *F. verticillioides*. The underlying mechanism behind this observation requires further investigation.

In our previous publication, we found that vacuoles were important for fumonisin biosynthetic enzyme localization and FB_1_ production. However, the vacuolar morphology was unaffected in Med1 LxxLL motif mutants as compared with wild-type M3125. In consideration of the vital roles of the LxxLL motif in the association between Med1 and various proteins [24,49], we analyzed the DEPs in FvMed1^ΔLxxLL^ and FvMed1^LxxAA^ strains under fumonisin-inducing conditions to elucidate the regulatory mechanism of the Med1 LxxLL motif in FB_1_ biosynthesis. Through proteomic profiling, we found that the Med1 LxxLL motif was involved in the regulation of mitochondrial functions and intracellular metabolism. Noticeably, several metabolic pathways, such as carotenoid biosynthesis, starch and sucrose metabolism, and thiamine metabolism, were significantly enriched in FvMed1^ΔLxxLL^ and FvMed1^LxxAA^. Therefore, we postulated that the Med1 LxxLL motif regulates fumonisin production by altering the metabolism of several key intermediates of FB_1_ biosynthesis, either directly or indirectly.

Maize starch consists of two key polymers, amylopectin and amylose. These two polymers have a unique set of macromolecular organizations and physical properties [55,56]. Shim and colleagues showed that maize endosperm, which contains high levels of starch, strongly supported *F. verticillioides* growth and FB_1_ production [42]. When inoculated with *F. verticillioides*, the highest levels of FB_1_ were detected in mature dent maize kernels, whereas significantly low levels of FB_1_ were detected in blister-stage maize kernels [43]. Since the starch composition is related to the kernel development stage, subsequent studies investigated the effect of individual starch components on FB_1_ biosynthesis in vitro. It was found that cultures containing amylopectin or dextrin as the sole carbon source produced higher FB_1_ than cultures provided with amylose or maltose [43]. In the current study, the expression of key genes involved in starch metabolism was significantly decreased in Med1 LxxLL motif mutants at both transcription and translation levels. Moreover, an accumulation of amylopectin was observed in the mycelia of Med1 LxxLL motif mutants. Considering these results, we propose that starch metabolism is closely associated with fumonisin biosynthesis, and the increased amylopectin content contributed to elevated FB_1_ production in Med1 LxxLL motif mutants.

Numerous studies have shown that oxidative stress is associated with mycotoxin biosynthesis [57,58,59]. In vitro experiments with liquid culture showed that treatment with pro-oxidant agents, such as H_2_O_2_ and diamide, significantly stimulated DON biosynthesis in *F. graminearum*, but the amount of DON production drastically decreased when catalase was added to the liquid medium [60]. Jayashree and Subramanyam found that oxidative stress was the prerequisite for aflatoxin biosynthesis in *A. parasiticus* [61]. Subsequent experiments revealed a correlation between the transcription level of aflatoxin biosynthetic genes and the activity of antioxidant enzymes [62]. As a group of well-studied antioxidants, carotenoids are part of the antioxidant defense system in humans and plants [63,64]. However, the effects of carotenoids on mycotoxin biosynthesis in plant pathogens have been scarcely studied. While earlier research showed that fumonisin accumulation was significantly lower in the high-carotenoid maize breed than the control maize breed [64], the inhibiting effects of carotenoids on fumonisin biosynthesis in *F. verticillioides* remain unknown. In the current study, we found that the expression of key genes involved in carotenoid metabolism was downregulated in Med1 LxxLL motif mutants. In addition, the knockout of carotenoid biosynthetic genes resulted in a significant reduction of carotenoid production, thereby leading to the enhanced expression of fumonisin biosynthetic genes and the accumulation of FB_1_ in *F. verticillioides*. These data indicated that the elevated FB_1_ production in Med1 LxxLL mutants was associated with decreased production of carotenoids.

## 4. Conclusions

In the present study, we identified the LxxLL motif of the Med1 protein in the mycotoxigenic fungus *F. verticillioides* and investigated its functions in fumonisin biosynthesis via multidisciplinary approaches. The results indicated that the Med1 LxxLL motif is important for fumonisin biosynthesis, which regulates FB_1_ production by affecting amylopectin and carotenoid production under fumonisin-inducing conditions. Our study provides a further illustration of the molecular mechanisms of the Med1 LxxLL motif in regulating fumonisin biosynthesis in the mycotoxigenic fungus *F. verticillioides*. Future research could focus on the binding targets of the Med1 LxxLL motif, which is involved in starch and carotenoid metabolism.

## 5. Materials and Methods

### 5.1. Strains and Culture Assays

*F. verticillioides* wild-type strain M3125 was used for the construction of the mutants generated in this study. All strains were grown at 28 °C on potato dextrose agar (PDA) plates for mycelial growth. Conidia production on the PDA plate was measured as described previously [34,35]. For the conidia germination assay, conidia harvested from PDA plates were cultivated on water agar (WA) plates at 28 °C for 7.5 h in the dark. For FB_1_ production and fumonisin biosynthetic gene expression assays, all strains were grown in Myro medium at 28 °C for 7 days and 2 days, respectively [34].

### 5.2. Strain Construction

All mutants were constructed following the homologous recombination protocol described previously [34,35]. The open reading frame of each gene was replaced with the Hygromycin B phosphotransferase gene (HPH). The gene deletion mutants ΔFvMed1 and ΔFvPSY-PDS were identified by PCR assays, and the null mutations were verified by qRT-PCR assays. The Med1 LxxLL motif mutants FvMed1^ΔLxxLL^ and FvMed1^LxxAA^ were further verified by DNA sequencing. All primers used are listed in Table S1.

### 5.3. Analysis of FB_1_ Production 

To measure FB_1_ production, M3125 and each mutant were grown in liquid Myro medium. After incubation at 28 °C for 7 days, 1 mL of Myro medium and all fungal mycelia of each strain were harvested, respectively. Subsequently, FB_1_ was assayed with a competitive ELISA detection plate kit (Aijude, Nanjing, China) following the manufacturer’s protocols. The experiments were performed three times independently with three replicates.

### 5.4. Microscopic Examinations

Vacuole morphology was observed with a Leica TCS SP5 confocal microscope (Wetzlar, Hessen, Germany). The wild-type M3125 and mutants ΔFvMed1, FvMed1^ΔLxxLL^, and FvMed1^LxxAA^ were cultured in YEPD for 1 day before staining with CMAC (KeyGen, Nanjing, China), a dye that labels the vacuole. The laser excitation wavelength was set at 353 nm for CMAC (blue fluorescence).

### 5.5. Proteomic Data and Bioinformatic Analysis

The mycelium samples were frozen in liquid nitrogen for protein extraction. The total protein of each sample was extracted by RIPA extraction buffer as described previously [65]. DEPs in wild-type and Med1 LxxLL mutants were obtained by 4D-label-free quantitative proteomics analysis. Proteins with an expression fold change >2 and *p* < 0.05 were filtered as differentially expressed. GO enrichment analysis was conducted by Blast2GO (https://www.blast2go.com/, accessed on 26 September 2023), and KGEE enrichment analysis was performed via KAAS (https://www.genome.jp/tools/kaas/, accessed on 26 September 2023). The experiment was performed by Shanghai Applied Protein Technology Co., Ltd. (Shanghai, China).

### 5.6. Quantification of Amylopectin Content and Carotenoid Production

The mycelia grown in Myro medium for 2 days were used for the measurement of amylopectin or carotenoid with an Amylopectin Assay Kit BC4270 (Solarbio, Beijing, China) or a Plant Carotenoid Content Assay Kit BC4330 (Solarbio, Beijing, China), respectively. Briefly, 0.1 g samples of nitrogen-ground mycelia were added to 1 mL of lysis buffer in the amylopectin or carotenoid detection kit. Subsequently, quantification of amylopectin content or carotenoid production was conducted following the manufacturer’s protocols. The experiments were performed three times independently with three replicates.

### 5.7. Effect of Med1 LxxLL Mutations on the Expression of Key Genes in Starch Metabolism and Carotenoid Biosynthesis

To measure the expression levels of key genes in starch metabolism and carotenoid biosynthesis pathways, mycelia were harvested from 2-day-old Myro cultures. The total RNA of each sample was extracted by the RNAsimple Total RNA Kit (Tiangen, Beijing, China) and used for cDNA synthesis with the HiScript II Q RT SuperMix for qPCR kit (Vazyme Biotech, Nanjing, China). The qPCR assays were conducted with a ChamQTM SYBR qPCR Master Mix kit (Vazyme Biotech, Nanjing, China). The GAPDH of *F. verticillioides* was used as the internal control. All primers used in this study are listed in Table S1. The relative quantification of each gene was calculated with the 2^−∆∆Ct^ method [66]. The experiments were performed three times independently with three replicates.

### 5.8. Data Analysis

Data on conidia production, conidia germination, FB_1_ production, gene expression, amylopectin, and carotenoid production of wild-type M3125 and its derived mutants were subjected to analysis of variance. Fisher’s LSD test was used to determine the differences (*p* < 0.05) among all treatments. All statistical analyses were conducted by Data Processing System (DPS) software 19.05 version.

## Data Availability

All data generated or analyzed in this study are included in this published article and its Supplementary information.

## Supplementary Materials

The following are available online at https://www.mdpi.com/article/10.3390/toxins15110652/s1, Table S1: Primers used in this study.
