# Supplementary material for: Characterization of Fusarium verticillioides Med1 LxxLL Motif Involved in Fumonisin Biosynthesis
Source: Toxins (Basel). 2023 Nov 13;15(11):652. doi: 10.3390/toxins15110652 (PMC10675092; doi:10.3390/toxins15110652)
Supplement: Supplementary file 1 [file toxins-15-00652-s001.zip › toxins-2660525-supplementary.pdf]

Table S1 Primers used in this study.

Primer	Sequence (5'-3')	Application
FVEG_02083 qF	TGATCCTGTGGCAGTCTTTG	For qPCR assay
FVEG_02083 qR	TCCGCGAGTGTAGTTGATTG	
FVEG_08584 qF	GGCCTGCTTTGGATGAAATG	For qPCR assay
FVEG_08584 qR	GTCAAGTCGGTTGGTGAGATAG	
FVEG_05521 qF	GGCTTCACCGTTGATCCTTAT	For qPCR assay
FVEG_05521 qR	CTGCTCGTTTGCGATGTAATG	
FVEG_09248 qF	TTCCGTTGGCGAAGGATAAG	For qPCR assay
FVEG_09248 qR	GTAAGGTGTCACAGCGTAGTAG	
FVEG_13055 qF	GAGTCCATCATAGGGCATCAAG	For qPCR assay
FVEG_13055 qR	CCAAAGTAAGGACGACGATAGG	
FVEG_03908 qF	ACAAGGTTGACGACGATGAG	For qPCR assay
FVEG_03908 qR	AAGGTAGCGGTGGAGTTAGA	
FVEG_04211 qF	CAACCTGCTCTTTGGTCTCT	For qPCR assay
FVEG_04211 qR	CCGTCAAGTTGTCCACAGTAT	
FVEG_16080 qF	CCAGGACATCATCACTCAGAAG	For qPCR assay
FVEG_16080 qR	TTATGGGCCGAACCAGAATC	
FVEG_06308 qF	AGAGGAGGATGTGCAGAAGA	For qPCR assay
FVEG_06308 qR	GTTGTTTGCGGTGTGGTTTAG	
FVEG_11351 qF	GGCGGTCTGGTCTTTATCTTTA	For qPCR assay
FVEG_11351 qR	CCACCAACATCCTTGCTATCT	
FVEG_04947 qF	GGTCAAGGGTAGTTCGTTCTT	For qPCR assay
FVEG_04947 qR	GGAGCGATGTTGGAGTAGTT	
FVEG_13849 qF	CCTTGGTACTTGGCTACTCTTG	For qPCR assay
FVEG_13849 qR	ACTGTGATGGAACCGATCTTG	
PSY_qF	GGTCTGACAGCATGGGATATT	For qPCR assay
PSY_qR	TAGGGTGAAAGGTCGGTTTG	
PDS_qF	CGCTGACCTTGTCTACACTTAC	For qPCR assay
PDS_qR	CGGACCAGTAGAAGGAGATACT	

FVEG_02675 qF	TCAACCGCCTCAAGAAGATG	For qPCR assay
FVEG_02675 qR	GTTGGCTCCATGAAGAGAGTAG	
Fum1_qF	GCTCTAGAGAACCGCACTATTC	For qPCR assay
Fum1_qR	TCAACTGGTACCGCCATATTC	
Fum6_qF	GACACAGAGACTGGGCAAATA	For qPCR assay
Fum6_qR	GAAACTCCCATGAGGCTAACA	
Fum8_qF	ATCACCGCCACTGTCTTTAC	For qPCR assay
Fum8_qR	GAAGCGTCGGACTTGATACTT	
Fum21_qF	GGAAAGCTAACGACGGAGATAC	For qPCR assay
Fum21_qR	GACTCTTCCGCTGATCCATTAC	
GAPDH_qF	CATCATCTCCAACGCCTCTT	For qPCR assay
GAPDH_qR	GTCATGAGACCCTCAACGATAC	
Med1_qF	GCTTCCGCACTCAGAAGAAA	For qPCR assay
Med1_qR	AAGGTGGCGACGAACATAAC	
Med1_LF/F	TCCCTAAGGATTTAGACTTC	Amplify FvMed1 5' flank sequence, for gene knock out
Med1_LF/R	ACCAGCCAGCCAACAGCTCCCGAGCTCCAGGTTGAAAGCAA	
Med1_RF/F	AATACGCAAACCGCCTCTCCCCGGCGGAGAAACTGAACGAC	Amplify *Fv*Med1 3' flank sequence, for gene knock out
Med1_RF/R	GCTTCCTCGCTGAGCACTT	
Med1_IN/F	CAGCCAACCAACACCAAGT	Amplify FvMed1 gene, for transformants screen
Med1_IN/R	GGCGATGCGGTGTTTACTT	
Med1_LF/F1	ACTTATTTATTAGCCTGGTTC	Transformants screen, for gene knock out
Med1_RF/R1	ATGTGCGAGTTCCTCAATG	
HPH-F	TTGGCTGGAGCTAGTGGAGGTCAA	Amplify *HY* fragment
HP-R	GTATTGACCGATTCCTTGCGGTCCGAA	
PH-F	GTTCCCGGTCGGCATCTACTCTAT	Amplify *YG* fragment
HPH-R	GATGTAGGAGGGCGTGGATATGTCCT	
LxxLL_LF/R	GCAAAGATCCTCCATATATTCATCTGCCCGCGTGAGTTCT	For Med1 LxxLL motif knock out
LxxLL_RF/F	GAATATATGGAGGATCTTTGC	

LxxLL_RF/R	CAATATCATCTTCTGTCGACGTGGACAGGGGACGACATT	
LxxLL_Down/F	TCTTGACGAGTTCTTCTGACAGCCTTTGTGACCTTGGG	
LxxAA_LF/R	ATCCTCCATATATTCTGCAGCCTTTCCCAAATCTGCCCGCGTGAG	For Med1 LxxLL motif amino acid substitution
LxxAA_RF/F	CTCACGCGGGCAGATTTGGGAAAGGCTGCAGAATATATGGAGGAT	
LxxAA_RF/R	ACCAGCCAGCCAACAGCTCCCTGTCCTTCGGATTATTCGTC	
LxxAA_Down/F	AATACGCAAACCGCCTCTCCCCTGTGGCGATGCTCTTTATG	
LxxAA_Down/R	GCTGATAAGGCTGTGGATG	
PSY_LF/F1	GTGAGTTTCGTGAAGTGCC	Amplify FvPSY 5' flank sequence, for FvPSY-PDS knock out
PSY_LF/R1	ACCAGCCAGCCAACAGCTCCCGACAGAGGTTTATTTATTTCG	
PDS_RF/F1	AATACGCAAACCGCCTCTCCCCCGAGCATTAGTTGGATTGGC	Amplify FvPDS 3' flank sequence, for FvPSY-PDS knock out
PDS_RF/R1	TAGGAGTCCAGCCGAGGTT	
PSY_LF/F2	TGATGGCTTGTTGAGACCC	Transformants screen, for FvPSY-PDS knock out
PDS_RF/R2	AGTCCACGAGAATCACCCA	
PSY_IN-R	GTCCAGTCCATCCCACAAG	
PDS_IN-F	CACTTTATCCACGCCCACT	
